# Medical Miscellany

**Published:** 1854-04

**Authors:** 


MEDICAL MISCELLANY.—Vaccination has been made compulsory in Egypt; in one year more than 79,000 children were vaccinated by the public officers.———Massachusetts has 1,406 physicians; being 1 to about 700 of the population.———The brain of the late Senator Atherton weighed 56½ ounces, or 7¼ less than Mr. Webster's and 7 more than Dupuytren's.———The deaths by small pox in N. York city last week were 43; but in the Kingdom of Hanover, where vaccination is compulsory, the number of deaths in a whole year was only 8, or 1 in every 5,728 of the population.———Dr. Mott of N. Y. City has lately been elected honorary member of the Medico-Chirurgical Society of London; Dr. John C. Warren of Boston is the only other honorary member in America.———A southern Doctor has been fighting a duel, but did not harm any body, his bullets being more innocent than his boluses.———The Mayor of Brooklyn, in his message, attributes three-fourths of the crime in that city to drunkenness.———Dr. Clarke, of Phila., replaced the severed part of a finger, half an hour after the accident, and it united fully.———Number of students in Jefferson Medical College 625; University of Penn., 510; Penn. Med. College 175, besides a Homeopathic, Female, Botanic, Eclectic and Independent Med. College in Philadelphia; University of Nashville has 240; University of Maryland 200; College Physicians and Surgeons, N. Y. has 250.———Oil of turpentine, applied by cotton, to a decayed tooth, is said to relieve pain.———A Yankee has invented a machine to take all the *lie* out of the quack advertisements, the truth being sifted out. But all the advertisements put into the hopper, up to date, have *entirely* disappeared!———The body of a man found buried six feet in guano, on the Island of Ichaboe, has been on exhibition at Baltimore. It is turned to a solid mass of stone, retaining all the minute outlines of a perfect gentleman.——— The Arabs in Upper Egypt cut up mummies and use them as fuel, to cook with, thus very improperly making light of a grave subject. ———Remove stains of Nitrate of Silver from the hand with a solution of Iodide of Potassium. Remove indelible ink from clothes by moistening a few moments with moist Chloride of Lime, forming Chloride of Silver, which dissolve out by aqua ammonia.

